# Pharmacological evaluation of vitamin D in COVID-19 and long COVID-19: recent studies confirm clinical validation and highlight metformin to improve VDR sensitivity and efficacy

**DOI:** 10.1007/s10787-023-01383-x

**Published:** 2023-11-13

**Authors:** Adel A. Gomaa, Yasmin A. Abdel-Wadood, Romany H. Thabet, Ghada A. Gomaa

**Affiliations:** 1 Department of Pharmacology, Faculty of Medicine, Assiut University, Assiut, Egypt; 2 Faculty of Agriculture, Assiut University, Assiut, Egypt; 3 Student Hospital, Assiut University, Assiut, Egypt

**Keywords:** Vitamin D, COVID-19, Long COVID-19, Observational studies, Randomized clinical trials, Genetic polymorphisms, Metformin

## Abstract

Nearly four years after its first appearance, and having gone from pandemic to endemic, the SARS-CoV-2 remains out of control globally. The purpose of this study was to evaluate the clinical efficacy of vitamin D (VD) in COVID-19 and long COVID-19, explain the discrepancy in clinical outcomes and highlight the potential impact of metformin on VD efficacy in recent articles. Articles from January 2022 to August 2023 were selected for this review. The objective of this study was achieved by reviewing, analyzing, and discussing articles demonstrating (1) the mechanism of action of VD (2) observational or randomized clinical trials (RCTs) that support or not the beneficial clinical effects of VD in COVID-19 or long COVID. (3) genetic and non-genetic reasons for the variation in the effects of VD. Articles were collected from electronic databases such as PubMed, Scopus, MEDLINE, Google Scholar, Egyptian Knowledge Bank, Science Direct, and Cochrane Database of Systematic Reviews. Twenty three studies conducted in vitro or in animal models indicated that VD may act in COVID-19 through protecting the respiratory system by antimicrobial peptide cathelicidins, reducing lung inflammation, regulating innate and adaptive immune functions and up regulation of autophagy gene activity. Our review identified 58 clinical studies that met the criteria. The number of publications supporting a beneficial clinical activity of VD in treating COVID-19 was 49 (86%), including 12 meta-analyses. Although the total patients included in all articles was 14,071,273, patients included in publications supporting a beneficial role of VD in COVID-19 were 14,029,411 (99.7%). Collectively, extensive observational studies indicated a decisive relationship between low VD levels and the severity of COVID-19 and mortality outcomes. Importantly, evidence from intervention studies has demonstrated the effectiveness of VD supplements in treating COVID-19. Furthermore, the results of 4 observational studies supported the beneficial role of VD in alleviating symptoms of long COVID-19 disease. However, eight RCTs and one meta-analysis of RCTs may contain low-grade evidence against a beneficial role of VD in COVID-19. Twenty-five articles have addressed the association between VDR and DBP genetic polymorphisms and treatment failure of VD in COVID-19. Impaired VDR signaling may underlie the variability of VD effects as non-genetic mechanisms. Interestingly, in recent studies, metformin has a beneficial therapeutic role in COVID-19 and long COVID-19, possibly by improving AMPK signaling of the VDR and enhancing the efficacy of the VD. In conclusion, evidence has been significantly strengthened over the past 18 months, with several meta-analyses and RCTs reporting conclusive beneficial effects of VD supplementation against COVID-19 and highlighting metformin to improve VDR sensitivity and efficacy in treating COVID-19 and long COVID-19.

## Introduction

Nearly four years after its first appearance, and after transition from a pandemic to an endemic phase, SARS-CoV-2 remains among the most troublesome respiratory viruses and is still out of control globally (Mahajan et al. 2023; WHO 2023). Furthermore, many studies published on long COVID indicate that in 50% to 70% of COVID-19 survivors may experience several post-COVID symptoms for up to 6 months which include a wide range of persistent health problems (Davis et al. 2023). The search for effective drugs for the treatment and prevention of coronavirus (COVID-19) is still underway. Numerous studies have shown potential for current therapies for prevention and treatment including antivirals, but the only clarity so far is that there is no effective drug driving clinical management in the WHO health emergencies programmer (Looi 2023). Furthermore, few studies attempt to investigate treatments for long post-COVID-19 syndrome for which there is no evidence of efficacy and little biological plausibility.

Extra-Skeletal functions of VD including differentiation and proliferation of cells, antioxidant, antibacterial, anti-inflammatory and immunomodulatory properties have been investigated in various tissues or cells by many investigators (Holick et al. 2023). Several epidemiologic studies have observed that low VD levels are found in a large percentage of COVID-19 patients with acute respiratory failure. Furthermore, it has been investigated that low levels of 25 hydroxyvitamin D are associated also with long COVID syndrome in survivors of COVID-19 (Filippo et al. 2023a). Vitamin D deficiency is widespread throughout the world, especially in southern European countries, and the Covid-19 virus has had a significant impact in these countries (Chiodini et al. 2021). Therefore, the use of VD supplements to prevent the spread of COVID-19 is a potential therapeutic strategy that is easy to implement (Cicero et al. 2022; Argano et al. 2023). VD works in more ways than one against COVID-19. Vitamin D interacting with its receptor (VRD)—Triggers the regulation of several genes involved in the immune system and enhances the innate and adaptive immune response against respiratory infections. In macrophages, it promotes the production of antiviral and antimicrobial proteins such as cathelicidins and beta-defensin-2 proteins that inhibit viral particle replication and promote removal of virus from cells by autophagy (Sartika and Gayatri. 2022). Analog calcitriol of vitamin D increased the expression of angiotensin-converting enzyme II (ACE2) in the lungs and alleviated acute lung injury (Xu et al. 2017). It also prevents cytokine storm and inflammatory processes in COVID-19 (Gilani et al. 2022; Bae et al. 2022).

Despite promising initial results, neither government agencies nor the World Health Organization has recommended incorporation of vitamin D into COVID-19 preventive or treatment guidelines. This could be possibly due to other studies found no such effects of VD (Brunvoll et al. 2022; Jolliffe et al. 2022).In addition many investigators attribute the reason for the contradiction in the results of the VD in the treatment or prevention of COVID-19 to the inaccuracy and heterogeneity with respect to design, drug dosage and population characteristics of the articles published in the first period after the outbreak of the pandemic (Jolliffe et al. 2022). Fortunately, the accuracy and heterogeneity regarding design, drug dose, and demographic characteristics of articles on COVID-19 treatment published in 2022 have been improved so far. In addition, several studies have observed that VDR gene polymorphisms may modulate response to VD therapy. The purpose of this study was to provide an up-to-date assessment of the evidence, evaluating the clinical efficacy of vitamin D in COVID-19, and long post COVID-19 and explaining discrepancy of clinical outcomes in articles published from January 2022 until now.

## Methods

Articles from January 2022 to August 2023 were selected for this review. Original research articles, whether experimental, observational, clinical trials or meta-analyses were included in this study. Articles published in languages other than English or published in a journal not indexed by Scopus were excluded from the review. The objective of this study was achieved by reviewing articles that elucidate (1) Mechanism of action of VD in treating COVID-19 in experimental studies (2) observational or randomized clinical trials that support the beneficial clinical effects of VD in COVID-19 (3) observational or RCTs have found no effect of VD in COVID-19 (4) Clinical studies support the beneficial effect of VD on long-COVID-19 (5) Genetic and non-genetic reasons for the differences in the effects of VD (6) potential impact of metformin on VD efficacy.

Qualitative and quantitative data were extracted from each study. Publication dates and number of included studies, patient characteristics, clinical status, sample size, VD supplementation and its effect on infection incidence, hospital stay, ICU admission rate, ventilation requirements, and mortality in COVID patients were extracted from the selected articles. Reports of published articles were collected from electronic databases such as PubMed, Scopus, MEDLINE, Google Scholar, Egyptian Knowledge Bank, Science Direct, and Cochrane Database of Systematic Reviews.

## Results and discussion

### Mechanism of action of vitamin D in treating COVID-19 in experimental studies

Twenty three studies conducted in vitro or in animal models on the mechanism of effect of vitamin D in COVID-19 infection were published from January 2022 to Aug. 2033.These studies indicate that vitamin D plays an important role in protecting the respiratory system through antimicrobial peptide cathelicidins, which have direct antimicrobial effects on bacteria, viruses, and fungi. In an in vitro study, calcitriol showed significant efficacy against SARS-CoV-2 in cell-based assays (Mok et al. 2023). They suggested that calcitriol acts by modulating the vitamin D receptor pathway to increase the expression of cathelicidin. It has effects on several innate immune mechanisms in the airway (Stapleton et al. 2022). The conflicting effects of calcitriol may be due to differences in vitamin D metabolism and the dose of calcitriol given to mice may have been too low to maintain adequate levels.

Meanwhile, Arora et al. (2022) investigated that high-dose VD reduced lung inflammation in mice but not hamsters. They observed faster recovery in VD-treated mice that survived SARS-CoV-2 infection. However, there was no action on gene expression of SARS-CoV-2 in the lungs of mice or hamsters. They observed that VD deficiency increased disease severity, while VD sufficiency or supplementation reduced inflammation after H1N1 and SARS-CoV-2 infection. Several investigators have also pointed to the reduction of inflammation by VD in COVID-19 infection as the main mechanism of VD action. In addition, other researchers observed that new biomarkers for inflammation such as the systemic inflammatory index and response were negatively associated with VD concentrations (Dziedzic et al. 2022).

The potential effect of VD in regulating the innate and adaptive immune function in SARS-CoV2 infection has been reviewed by several investigators (Bikle 2022). Regarding innate immune systems, it modulates constitutive expression of recognition receptors such as TLRs to identify SARS-CoV2. It promotes the production of antimicrobial peptides such as cathelicidins and b-defensins from neutrophils, macrophages and from epithelial respiratory cells which stimulate clearance of these viruses. Importantly, the attenuating effect of VD on chronic activation of innate immunity that results in a cytokine/bradykinin storm. It works by down regulating TLRs and increasing IL-10 production by regulatory T cells, while inhibiting Th17 cells and the TNF/NFκB and IFNγ signaling pathways. The active metabolite, 1, 25(OH) 2D, regulates adaptive immunity by increasing the production of virus-specific IgG1 antibodies. However, it reduces DC maturation and regulation of key transcription factors such as STAT3 and STAT3 activator of IL-6 transforming T cell. Therefore, it inhibits inflammatory processes (Bikle 2022). Briceno Noriega and Savelkoul (2022) confirmed that VD plays an important role against the endemic phase of COVID-19 respiratory tract infection by acting as an immune modulator. It would play a protective role in the endemic phase of COVID-19 by stimulating the cellular receptor angiotensin converting enzyme 2 (ACE2)/Ang (1–7)/Mas G and inhibiting the expression of renin and the angiotensin II receptor type I (AT1R) axis. Moreover, cathelicidin LL-37 and human-defensin 2 induced by 1, 25(OH) 2D interact with the SARS-CoV-2 spike protein and inhibit viral binding to ACE2 (Pouremamali et al. 2022). Laboratory evidence of clinical studies has also shown that VD plays an immunomodulatory role during COVID-19 infection in preventing hyperinflammatory conditions associated with COVID-19(Sharif-Askari et al. 2022).

Cimmino et al. (2022) hypothesized that VD prevents IL-6 deleterious effects in COVID-19 infection. IL-6 induces COVID-related thrombosis via endothelial dysfunction with tissue Factor and adhesion molecules expression up regulation and ACE2r. Moreover, it was observed that the expression of VDR was statistically lower in patients with COVID-19 than healthy subjects and that the level of IL-6 was statistically higher in the COVID-19 group. Therefore, patients with severe COVID-19 may benefit from vitamin D supplementation, which would help reduce the production of IL-6 that causes a cytokine storm and thus reduce the severity of the disease. (Azmi et al. 2023; Chileshe et al. 2022; Holick et al. 2023). Furthermore, calcitriol has been shown to improve the barrier function of 16HBE cell layers based on two independent measures by inhibiting TNF-α-induced barrier leakage of epithelial cells in a human lung culture model (Rybakovsky et al. 2023).

**Fig. 1:**
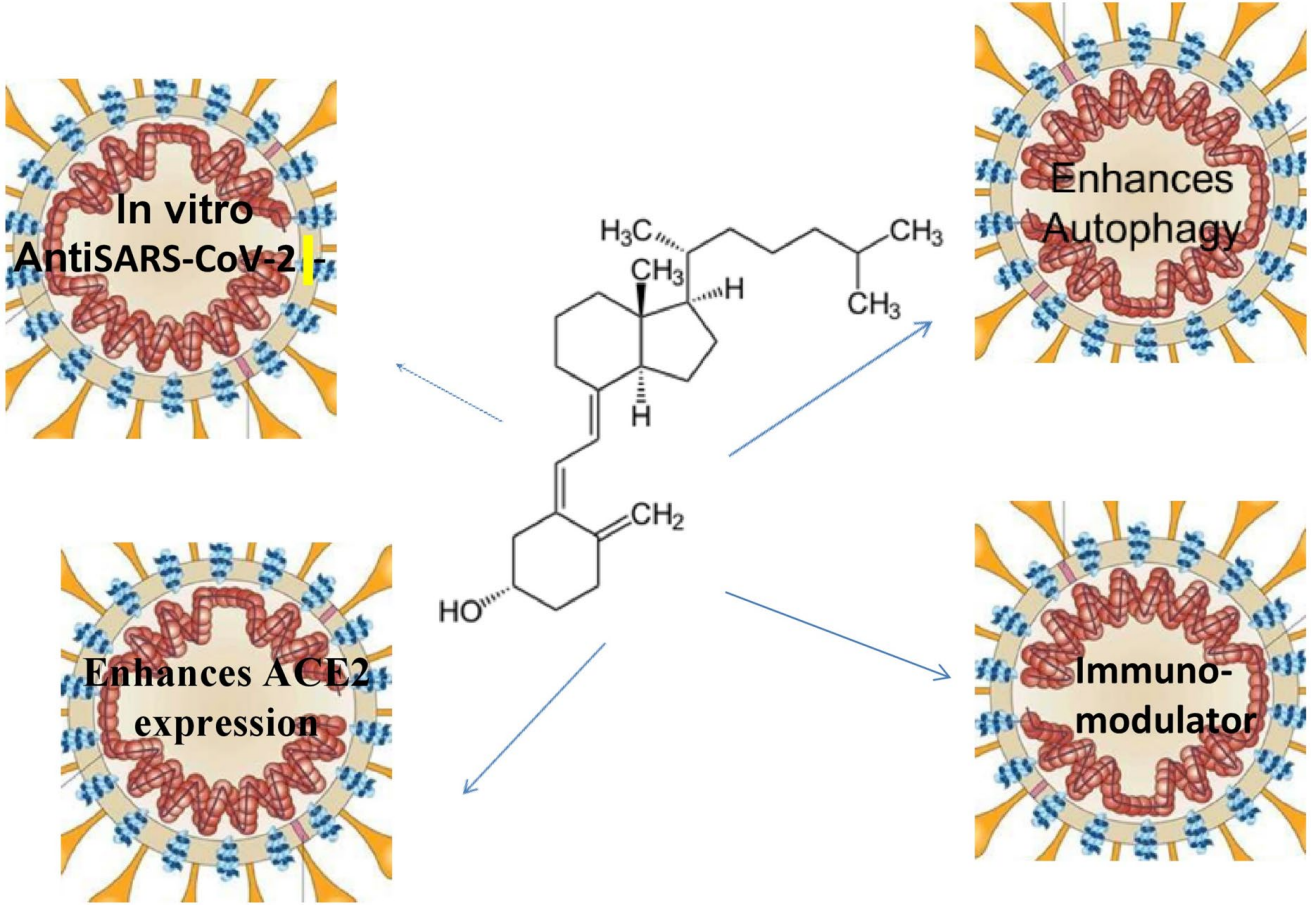
Proposed mechanisms of vitamin D in COVID-19. Vitamin D can act through antiviral action, immunomodulation, and promotion of autophagy and ACE2 expression

The mechanism of action of VD in COVID-19 diseases may also be through regulation of autophagy gene activity by VD/VDR. VD promotes autophagy via genomic or nongenomic signaling pathway to regulate a wide range of functions of many organs (Sartika and Gayatri 2022). By activating autophagy, VD protects various organs from oxidative stress and apoptosis and regulates immune modulation, cell proliferation and differentiation, and control of inflammation. Furthermore, VD supplementation can enhance autophagy to prevent many human diseases as a part of human homeostasis mechanism (Bhutia et al. 2022). In vivo and in vitro studies, VD3 supplementation was able to activate autophagy and in vitro significantly enhances gene expression of VDRs and autophagy (Chen et al. 2022) (Fig. 1).

## Clinical studies of Vitamin D in COVID-19

For this study, 58 publications published from January 2022 to August 2023 that met the eligible criteria were selected. Number of publications supporting the beneficial clinical effect of VD in treating COVID-19 is 49 (86%) including 12 meta-analyses. Overall 14,071273 patients were included in these publications while 14,029,411 (99.7%) patients were included in publications supporting the beneficial clinical effect of VD in COVID-19. Publications are discussed under three headings, observational studies, RCTs (intervention) that support a beneficial clinical effect of VD, and clinical studies that disapprove of the use of VD in the treatment of COVID-19.

### Observational studies

Although there is a small number of conflicting evidence from interventional studies on the benefits of VD supplementation in COVID-19 patients, these epidemiological observational studies collectively confirm the association of low levels of vitamin D with COVID-19 susceptibility, severity, and mortality outcomes. In this study, 29 observational studies were published from January 2022 to August 2023, confirming the previous conclusion. In a very large population-based meta-analyses study by Petrelli et al. (2023), including 74 articles, 27 meta-analyses noted that vitamin D3 deficiency or insufficiency is associated with increased risk of SARS-CoV-2 infection, COVID-19 severity, and mortality risk, with highly suggestive evidence. Equally important, the results of a large number of observational groups, a retrospective case–control study published by Israel et al. in 2022, showed that there is an inverse relationship between the level of VD and the risk of SARS-CoV-2 infection and disease severity in infected patients. Moreover, in a prognostic association study, vitamin D deficiency contributes to an increased risk of severe outcomes of COVID-19, which was evident in a retrospective observational study conducted on 2342 patients with COVID-19 in a clinical hospital of infectious disease (Toban et al. 2023) and also in a retrospective observational cohort study including 2908 patients(Ramirez-Sandoval et al. 2022). These and 18 other studies suggest that low VD may be a risk factor for poor prognosis among patients admitted for COVID-19, therefore, serum levels of 25(OH)D for COVID-19 patients can be used as an independent indicator of prognosis (Table1).

**Table 1:** Characteristics of observational clinical studies supporting the use of vitamin D in the treatment and prevention of COVID-19 in articles published from January 2022 to Aug. 2023

Design of study	Participants	Dose of VD	Duration of use	Primary outcomes	References
Observational Clinical trial	140 COVID-19 patients participated in this study (65 outpatients and 75 inpatients). .25(OH)D levels was measured	No intervention,	No	− 25(OH)D levels were inversely correlated with TNFa, TNFa mRNA, IL-6, and D-dimer levels—The lower mortality and severity of COVID-19 patients was with higher 25(OH)D levels	Beheshti et al. 2023
Observational cohort study	288 patients	2 boluses of calcifediol of 450 µg each	for 2 consecutive days for a total of 900 mcg	The percentage of deaths was significantly higher in patients who did not receive calcifediol Longer duration of hospitalization in patients with severe 25OHD deficiency This study demonstrated that best prognosis of COVID-19 patients with adequate vitamin D levels and patients treated with calcifediol supplementation	Mingiano et al. 2023
Meta-analyses	74 Studies, 27 meta-analyses, 12,767,045 patients	Various	Various	Low levels of vitamin D3 were associated with significant risk of severity,mortality compared to patients with sufficient levels. Vitamin D3 supplementation, was associated with significantly reduced infection, mortality, infection severity	Petrelli et al. 2023
Prospective cohort study observational study	191 patients	No intervention	No intervention	Lower serum 25(OH)D levels were significantly associated with an increased number of,the severity of COVID, mechanical vent. However, patients with either sufficient or insufficient vitamin D levels did not develop mortality	Rachman et al. 2023
A case–control study Observational study	46 patients	No intervention	No	This study showing an association between low serum vitamin D and ARTIs including COVID-19	Elmi et al. 2023
Retrospective observational study, Poland,	134 healthy subjects, assessing the Incidence of infection after one year	No intervention	No	Increased risk of COVID-19 infection was only observed in those with severe 25OHD deficiency < 12 ng/mL	Basińska-Lewandowska et al. 2023
Retrospective study observational study	Fifty Long-COVID and 50 non-Long-COVID subjects	No intervention	–	COVID-19 survivors with Long-COVID have lower 25(OH)vitamin D levels as compared to matched-patients without Long-COVID	di Filippo et al. 2023a
Prospective study Observational study	A total of 73 COVID-19 patients and 30 control subjects were enrolled in this study	No intervention		25(OH)vitamin D levels at hospital-admission strongly predicted the occurrence of worsening outcomes in COVID-19	di Filippo et al. 2023b
Retrospective study observational study	MS-COVID, *n* = 149 MS-NCOVID, *n* = 292	No intervention		Study reinforces the importance of supplementation of vitamin D levels in PwMS	Montini et al. 2023
Retrospective observational study at the Clinical Hospital of Infectious Diseases	2342 COVID-19hospitalized A total of 1194 patients were with vitamin D deficiency ≤ 20 ng/mL of 25(OH)D	No intervention		Vitamin D deficiency was associated with disease severity and death outcome in hospitalized COVID-19 patient	Topan et al. 2023
Center-based Observational prospective study	93,685 participants Serum level & clinical outcomes	No intervention,		Higher vitamin D intake was associated with decreased risk of ischemic stroke and pneumonia mortality	Nanri et al. 2023
A retrospective observational study	83 patients	No intervention		The inverse relationship between Vitamin D level and poor composite outcome suggests that low Vitamin D may be a risk factor for poor prognosis among patients admitted for COVID-19	Tan et al. 2023
Meta-analyses of observational studies	149, 865 participants	No intervention		Evidence confirms that vitamin D reduces respiratory cancer mortality, All-cause mortality is decreased in COVID-19 patients	Cao et al. 2023
A longitudinal, real-world observational cohort study	Total of 161 cases	No intervention		Vitamin D status may have effects on the progression and resolution, but not the onset of Delta variant-induced pneumonia in adults	Huang et al. 2023
Observational study	742 patients admitted to the post-COVID-19 outpatient service	No intervention		Vitamin D deficiency is frequent in COVID-19 survivors, especially in older adults. Low vitamin D levels are associated with poor physical performance, in particular in old age	Galluzzo et al. 2022
Single center observational prospective study	310 critically ill COVID-19 patients, aged ≥ 65 years	No intervention,		Vitamin D deficiency was associated with an increased risk of delirium and mortality among critically ill elderly COVID-19 patients	Gholi et al. 2022
Observational study	447 adults	No intervention	No intervention	Low levels of 25(OH)D were associated with a higher risk of severe COVID-19	Nielsen et al. 2022
Prospective observational study	232 patients	No Intervention		Unvaccinated Caucasian adults with a low vitamin D state have higher mortality due to SARS CoV-2 pneumonia, which is not explained by confounders	Barrett et al. 2022
Retrospective cohort observational study	220,265 patients supplemented with vitamin D_3,_ 34,710 supplemented with vitamin D_2_, and 407,860 untreated patients	Dosage options included 20 IU, 40 IU, 100 IU, 125 IU, 200 IU, 250 IU, 400 IU, 500 IU, 800 IU, 1000 IU, 2000 IU, 5000 IU, 8000 IU, and 50,000 IU	UP to 30 days	Vit. D_3_ and D_2_ supplementation reduced the associated risk of COVID-19 infection and death within 30-days by 33% Higher cumulative dosages and higher average daily dosages had a greater associated reduction in COVID-19 infection rates than lower dosages	Gibbons et al. 2022
Retrospective observational study	1176 patients	No intervention		Among hospitalized COVID-19 patients, pre-infection deficiency of vitamin D was associated with increased disease severity and mortality	Dror et al. 2022
Cohort observational study	131 adults with a positive SARS-CoV-2 and 18 adults with no COVID-19	No intervention		Lower Vitamin D levels are associated with severe disease	Baxter et al. 2022
Retrospective observational study	300 hospitalized covid-19 patients	No intervention		Low vitamin D levels are related to exaggerated inflammatory response, disease severity, and poor clinical outcome in hospitalized covid-19 patients	Mostafa et al. 2022
Retrospective observational study	16,446 (26.3%) COVID-19 patients and 46,005 (73.7%) negative control group patients	No intervention		Significant association between the suboptimal serum vitamin D level and COVID-19 infection	AlYafei et al. 2022
Retrospective observational study	165 women in the third-trimester of pregnancy	No intervention		These results show the relationship between vitamin D deficiency in pregnant women and the severity of COVID-19 infection	Vásquez-Procopio et al. 2022
Retrospective observational cohort study	2,908 patients	No intervention		Very low 25-hydroxyvitamin D levels measured at hospital admission were significantly associated with in-hospital mortality and are a useful prognostic biomarker in severe COVID-19 patients	Ramirez-Sandoval et al. 2022
large observational population study, a retrospective case–control study	464,393 Positive SARS-CoV-2 & control	No intervention		A significant association between vitamin D deficiency and the risks of SARS-CoV-2 infection and of severe disease in those infected patients	Israel et al. 2022

A large pharmacoepidemiological study (220,265 patients) indicated that VD3 and VD2 supplements reduced the risks associated with COVID-19 infection by 20% and 28%, and mortality by 33% and 25%, respectively. Furthermore, patients with initially low VD levels benefited more from VD supplementation than did patients with a higher serum level. This study also suggested that patients receiving higher bolus doses and higher daily doses had significantly reduced rates of COVID-19 infection compared to patients receiving lower doses with similar levels of VD (Gibbons et al. 2022). Another canter-based observational prospective study (93,685 patients) demonstrated that higher VD intake was associated with decreased risk of ischemic stroke and pneumonia mortality (Nanri et al. 2023). Interestingly, in an observational cohort study, two doses of calcifediol 450 mcg each for two consecutive days reduced the percentage of mortality with the best prognosis in COVID-19 patients significantly higher than those who did not receive calcifediol. (Mingiano et al. 2023). Furthermore, VD levels were inversely correlated with inflammatory markers as TNFa, TNFa mRNA, IL-6, and D-dimer levels with lower mortality and severity of COVID-19 at higher VD levels (Beheshti et al. 2023). In general, extensive observational studies indicate a decisive relationship between low serum VD levels and mortality outcomes. However, the results of cohort studies generally suffer from insufficient follow-up time, completeness of follow-up, and the number of influencing factors cannot be well controlled. Therefore, correlation in observational studies cannot be equated with causation in actual RCTs.

### Randomized controlled trials

Due to the characteristics of RCT, strict patient exclusion and inclusion criteria lead to limitations in the representativeness bias and external validity of the research results. RCTs of VD supplementation in COVID-19 are necessary to conclusively demonstrate benefit. As shown in Table 2, in this study, the scale of patients enrolled in 11 RCTs was 297,494 and in 11 meta-analyses of RCTs was 296,087 which were published from January 2022 to August 2023. Although different doses of VD have been used to test the efficacy of VD against COVID-19, all studies confirm the clinical safety of high-dose VD supplementation and the clinical benefit of VD in COVID-19. However, high-dose supplementation regimens had a significantly better clinical outcome compared to lower doses ( Sarhan et al. 2022; Cervero et al. 2022; Cicero et al. 2022; Shah et al. 2022; Annweiler et al. 2022; Tentolouris et al. 2022; Torres et al. 2022; Menger et al. 2022; Asla et al. 2023). Moreover, parenteral high dose was associated with a greater impact on reduction of mortality (Menger et al. 2022; Sarhan et al. 2022; Zaazouee et al. 2023; Asla et al. 2023). Additionally, VD may have greater benefit if given early in mild to moderate COVID-19 cases. In a randomized, double-blind, parallel trial enrolling highly exposed workers from four hospitals in Mexico City, a small daily dose (4000 IU) of VD supplementation prevented SARS-CoV-2 infection without serious complications and regardless of serum VD level. (Villasis-Keever et al. 2022).

**Table 2:** Characteristics of randomized controlled (intervention) clinical studies supporting the use of vitamin D in the treatment and prevention of COVID-19 in articles published from January 2022 to Aug. 2023

Design of study	Participants	Dose of VD	Duration of use	Primary Outcomes	References
case‐control study	200 critically ill patients with COVID‐19 aged 35 − 85 year, who were hospitalized in the ICU	10,000 IU/day vit D	30 days	Vitamin D supplementation in critically ill patients with COVID‐19 has the potential to increase survivability within the first 30 days of hospitalization	Gholamalizadeh et al. 2023
meta-analysis of randomized controlled trials	Nine RCTs with 1586 confirmed COVID-19 patients	Various, single IM of 200,000 units of D3, Oral calcifediol in soft capsules (0.532 mg) on days 3 and 7, and then weekly until discharge	Various one day to two months	Vitamin D reduced the risk of ICU admission and showed superiority in changing vitamin D level compared to the control group	Zaazouee et al. 2023
Meta-Analysis and Trial Sequential Analysis	five RCTs, 1400 patients	Various, 5.000 IU/day to 100.000 IU/day	Various, 2–4 weeks	Vitamin D administration results in a decreased risk of death and ICU admission	Argano et al. 2023
Meta-Analysis	A total of 8001 COVID-19 patients from 42 studies were included	Various, from single IM high dose to 50 000 to 100 000 IU which had a significantly better clinical outcome compared to lower and higher doses	Various from single dose to daily use for 4 weeks	Patients took Vit-D supplements had a significantly lower mortality rate, hospitalization duration, ICU admission rate, and mechanical ventilation rate than those who did not -High dose had a significantly better clinical outcome compared to lower	Asla et al. 2023
A Randomized Clinical Trial	116 patients	Alfacalcidol 1 mcg/day or cholecalciferol 200,000 IU IM	Minimum of five days	High-dose vitamin D was promising treatment in the suppression of cytokine storms among COVID-19 patients and was associated with better clinical improvement and fewer adverse outcomes compared to low-dose vit. D	Sarhan et al. 2022
A double-blind, parallel, randomized trial	321 Frontline healthcare workers from four hospitals in Mexico City, who tested negative for SARS-CoV-2 infection	Participants received 4,000 IU VD (VDG) or placebo (PG) daily	30 days	SARSCoV-2 infection rate was lower in VDG than in PG. the results suggest that VD-supplementation in highly exposed individuals prevents SARS-CoV-2 infection without serious AEs and regardless of VD status	Villasis-Keever et al. 2022
Multicenter, single-blinded, prospective randomized pilot clinical trial (RCT)	87 participants	Daily oral high dose of cholecalciferol (vitamin D3) (10,000 IU/day) in comparison with a moderate dose of D3 (2000 IU/day)	14 days	Higher dose of vitamin D3 may be effective to improve the oxygen requirements during hospitalization by COVID-19 and helping to improve the prognosis during the recovery	Cervero et al. 2022
A meta-analysis	23 studies, including 2692 SARS-CoV-2 patients	Various	Various	Plasma 25-OH-vitamin D deficiency is associated with an increased risk of developing severe SARS-CoV-2 disease. treatment with high-dose vitamin D was associated with a reduced risk of COVID-19 mortality	Cicero et al. 2022
Meta-analysis	3 to 13. Meta-analysis of seven systematic Reviews including 48,458	Various	Various	This study shows that vitamin D supplementation is effective in reducing the COVID-19 severity. Calcifediol at a high dose helped significantly reduce ICU admissions	Shah et al. 2022
A multicenter, open-label, randomized controlled **o**f 9 medical centers in France	254 met eligibility criteria	Single oral high-dose (400,000 IU) or standard-dose (50,000 IU) cholecalciferol administered	72 h after the diagnosis Follow up at 14 days	Early administration of high dose versus standard-dose vitamin D3 to at-risk older patients with COVID-19 improved overall mortality at day 14	Annweiler et al. 2022
A randomised, placebo-controlled, study (SHADE study)	Forty SARS-CoV-2 RNA positive individuals	Patients with vitamin D deficiency were receive daily 60,000 IU cholecalciferol (5 ml oral solution in nano droplet form)	For 7 days	Greater proportion of vitamin D-deficient individuals with SARS-CoV-2 infection turned SARS-CoV-2 RNA negative with a significant decrease in fibrinogen on high-dose cholecalciferol supplementation	Rastogi et al. 2022
systematic review and meta-analysis	38 eligible studies, with one endpoint, including two RCT and 27 cohort studies 205,565 patients	Various	Various	Supplementation was associated with a significant lower risk of both Covid-19 severe disease and mortality	D'Ecclesiis et al. 2022
In vitro and in the setting of COVID-19 hospitalized patients, investigation study	treated (43 patients), or not (37 patients), with vitD	administrated as 50,000 IU	weekly for 2–3 weeks	VitD could augment signaling of RIG-1/MDA-5 and IFN α/β pathways and VitD supplementation could help reduce the severity of COVID-19 disease by boosting innate immunity of patients	Hafezi et al. 2022
Meta-Analysis	Twenty-three studies 1548 (873 intervention: 675 control)	Various , from boluse 200,000 IU, 80,000 IU, and 300,000 IU or two doses of 200,000 IU for 2 successive or 60,000 IU/day for 8–10 days	Various, day for 8–10 days	Vitamin D supplementation had no significant impact on the risk of COVID-19 infection, whereas it showed protective effects against mortality and ICU admission in COVID-19 patients	Hosseini et al. 2022
Meta‐analysis and meta‐regression	2078 patients from nine studies (583 received vitamin D supplementation, while 1495 did not)	vitamin D is concerned, ranging from low daily doses like 1000 IU of cholecalciferol to high‐dose boluses like 400,000 IU of cholecalciferol	Various	Indicates a beneficial role of vitamin D supplementation on ICU admission, but not on mortality, of COVID‐19 patients Higher dose is better	Tentolouris et al. 2022
Meta‐analysis and meta‐regression	11 studies with 22,265 Covid‐19 patients	Vitamin D doses were administered orally dosage varied, ranging from 25,000 IU/month up to 200,000 IU/day for two consecutive days	Daily for two days	D supplementation was associated with reduction in intensive care unit admission rate reduction of the need for mechanical ventilation and reduction of mortality from Covid‐19	Hariyanto et al. 2022
Randomized, double blind and placebo controlled	50 subjects	Vitamin D 25,000 IU per day	Daily for 4 days, then 25,000 IU per week up to 6 weeks	Vitamin D significantly reduced the duration of supplemental oxygen among the patients who needed it and significantly improved the clinical recovery of the patients, as assessed by the WHO scale	De Niet et al. 2022
Cohort intervention clinical trial	Patients with severe COVID19 who had been treated (20 patients), or not (25 patients), with VitD, during their stay in the intensive care unit	(50,000 IU of cholecalciferol weekly	3 weeks	Vit. D reduced levels of STAT3, JNK and AKT pathways and lower levels of proinflammatory cytokines such as IL-6, IL-17, and IL-1β were observed in VitD patients, CRP, - shorter length of ICU stay	Sharif-Askari et al. 2022
A multicenter, single-blind, prospective, randomized clinical trial	85 patients,,41 patients received the supplementation of 10,000 IU/day (high dose) and 44 patients received 2000	10,000 IU/day of cholecalciferol (vitamin D_3_) in comparison with 2000 IU/day	14 days	High doses of vitamin D_3_ improve the inflammatory against pseudotyped SARS-CoV-2 infected cells, shortening the hospital stay and, possibly, improving the prognosis	Torres et al. 2022
Randomized clinical trial	129 patients were randomized. Group I (n = 56) received a bolus of cholecalciferol Group II (n = 54) did not receive the supplementation	Bolus of cholecalciferol at a dose of 50,000 IU	The first and the eighth days of hospitalization	The serum 25(OH)D level on the ninth day was negatively associated with the number of bed days and neutrophil Lymphocyte counts were significantly higher while the C-reactive protein level was significantly lower on the ninth day of vitamin D sup	Karonova et al. 2022
Meta‑analysis of randomized controlled trials	Sixteen randomized clinical trials with 2449 patients	Various	Various	Vitamin D administration was associated with lower overall mortality. Parenteral administration might be associated with a greater impact on mortality	Menger et al. 2022

Several meta-analyses compare the effectiveness of low-dose or high-dose on the clinical severity of COVID-19 (Shah et al. 2022; Cicero et al. 2022; Cervero et al. 2022; D'Ecclesiis et al. 2022; Hariyanto et al. 2022; Zaazouee et al. 2023). Anweiler et al. (2022) suggested that early administration of high dose (400,000 IU) versus standard dose (50,000 IU) of cholecalciferol to at-risk patients with COVID-19 reduced overall mortality. Likewise, Hosseini et al. (2022) meta-analysis found no change in the incidence of COVID-19 infection after VD supplementation, while showing protective effects against mortality and ICU admission in COVID-19 patients.The only meta-analysis with different results was published by Tentolouris et al. (2022). They suggested that daily oral doses as small as 1,000 IU of cholecalciferol to high doses as 400,000 IU of cholecalciferol have a beneficial role on ICU admission, but not on mortality. In cohort intervention clinical trial, vitamin D reduced markers of inflammation and shortened the length of stay in the intensive care unit. Vitamin D also led to decreased levels of STAT3, JNK, and AKT pathways, and decreased levels of proinflammatory cytokines such as IL-6, IL-17, and IL-1β (Sharif-Askari et al. 2022; Hafezi et al. 2022). Collectively, the evidence from these intervention studies has demonstrated clear efficacy of VD supplementation in treating COVID-19. This evidence has been significantly strengthened over the past 18 months, with several meta-analyses reporting conclusive, specific, and indisputable protective effects of VD supplementation against admission of COVID-19 patients to the ICU(D’Ecclesiis et al. 2022; Hariyanto et al. 2022; Cicero et al. 2022; Argano et al. 2023; Petrelli et al. 2023; Asla et al. 2023).

### Clinical studies have not found a beneficial clinical effect for vitamin D in treating COVID-19

Eight randomized control trials (RCT) and one met a-analysis of RCTs published from January 2022 to Aug. 2023 did not approve the use of vitamin D in the treatment and prevention of COVID-19., The scale of patients enrolled in these studies was 41,862. All randomized controlled trials confirm the clinical safety of high doses of vitamin D supplements, however, benefit has not been observed in any of the COVID-19 outcomes including length of hospital stay, disease incidence, number of days on respiratory support, mortality, admission to Intensive care unit, and prognosis. Four randomized controlled trials(44% of studies) evaluated the effect of a single oral bolus dose of cholecalciferol (100,000–500,000 IU) on length of hospital stay and respiratory deterioration, and found no effect in 649 patients with insufficient vitamin D level (Cannata-Andía et al. 2022; Mariani et al. 2022; Jaun et al. 2023; Abroug et al. 2023). Eight cohort studies and eight randomized controlled studies involving 3359 patients with COVID-19 were included in the only meta- analysis study (Zhang et al. 2023). This study showed that the results of the pooled analysis of cohort studies indicated that VD supplementation had a significant effect on reducing mortality in COVID-19 patients, while the results of the pooled analysis of RCTs showed that VD supplementation did not significantly change the mortality rate (Table 3).

**Table 3:** Characteristics of clinical studies do not approve the use of vitamin D in the treatment and prevention of COVID-19 in articles published from January 2022 to Aug. 2023

Design of study	Participants	Dose of VD	Duration of use	Primary outcomes	References
Multicentre randomized controlled clinical Trial	218 adult with Baseline of D3 were 32.5 ng/ml and 30.5 ng/ml for the vitamin D3 & placebo group, respectively	Single oral dose of 500 000 IU of vitamin D3 soft gel capsules (5 capsules of 100 000 IU)	once	Supplementation with a single, high dose of vitamin D3 at admission to patients hospitalized with mild-to-moderate COVID-19 did not prevent respiratory worsening as compared with placebo	Mariani et al. (2023)
Multicenter, randomized, placebo-controlled double-blind trial	40 patients	Single high dose of oral (140,000 IU, D3	followed by 800 IU daily	The intervention with 140,000 IU vitamin D3 + TAUdid not significantly shorten the length of hospital stay but was effective and safe for the elevation of serum 25(OH)D3 levels	Jaun et al. (2023)
Randomized controlled, parallel-group, blinded, clinical trial	A total of 117 patients	VDs (200,000 IU/1 ml of cholecalciferol (1 ml) oral form	Single dose	VDs was not associated with a shortened recovery delay when given to patients for whom the RT-PCR remained positive on the 14th day	Abroug et al. (2023)
Meta-analysis	Eight randomized controlled trials (RCTs) and eight cohort studies were included, involving 3359 COVID-19 patients	Heterogeneous	Various	The pooled analysis of randomized controlled trials showed that vitamin D supplementation does not have a significant impact on reducing mortality, ICU admission, and the rates of mechanical ventilation or intubation among COVID-19 patients	Zhang et al. (2023)
Single center, open label randomized clinical trial	155	received 10,000 IU of cholecalciferol daily orally	14 days	The daily supplementation of vitamin D in severe COVID-19 patients admitted to the ICU did not seem to reduce the number of days on respiratory support	Domazet Bugarin et al. (2023)
Three arm, parallel, randomised controlled trial	3100 participants	800 IU/day- or3200 IU/day vitamin D	six month supply	Supplementation with 800–3200 IU/day was safe and effective in increasing 25(OH)D concentrations, however, neither of the vitamin D doses had any effect on incidence of covid	Jolliffe et al. (2022)
Multicenter, randomized, placebo-controlled double-blind trial	80 (40 patients received Vit. D	140,000 IU vitamin D3	6 days	The intervention with 140,000 IU vitamin D3 + TAU did not significantly shorten the length of hospital stay	Jaun et al. (2023)
Quadruple blinded, randomised placebo controlled trial	Intervention *n* = 17,278) placebo (*n* = 17,323)	5 mL/day of cod liver oil (10 µg of vitamin D	Six month	Supplementation with cod liver oil in the winter did not reduce the incidence of SARS-CoV-2 infection, serious covid-19, or other acute respiratory infections compared with placebo	Brunvoll et al. (2022)
Multicentre, international, randomised, open label, clinical trial	Patients were randomised to receive a single oral bolus of cholecalciferol (*n* = 274) or no(*n* = 269	Oral bolus of cholecalciferol (100,000 IU)	Single	Administration of an oral bolus of 100,000 IU of cholecalciferol at hospital admission did not improve the outcomes of the COVID-19 disease	Cannata-Andía et al. (2022)

In a large-scale, quadruple-blind, randomized controlled trial (34,601 patients), 10 mcg of vitamin D daily for six months in the winter did not reduce the incidence of SARS-CoV-2 infection and severe COVID-19 outcomes compared with placebo(Brunvoll et al. 2022). Similar conclusions were achieved in three arm, parallel, randomised controlled trial observed that 800 IU/day or3200 IU/day vitamin D for six months had no effect on incidence of covid-19(Jolliffe et al. 2022). However, the overall number of randomized controlled trials that failed to find a beneficial role for vitamin D was small with a small sample size of the enrolled population and heterogeneous with respect to study design, dosing and intervention strategies. In addition to the conflicting results in the meta-analysis as described with Zhang et al. (2023). Therefore, these studies are considered to have low-grade evidence against the beneficial role of vitamin D in COVID-19.

## Impact of vitamin D on long COVID-19 syndrome

Two years after the SARS-CoV-2 virus began spreading globally, reports from most parts of the world indicate that a significant proportion of people who have recovered from COVID-19 have various health problems referred to as “long COVID-19.”. Several published studies on long COVID suggest that in 50% to 70% of COVID-19 survivors suffer from post-COVID symptoms for more than 3 months after acute disease (Fernández et al. 2021). Furthermore, other studies suggest that individuals may remain symptomatic months after initial recovery and an estimated 65 million or more people are living with the effects of long COVID-19 (Davis et al. 2023). long COVID-19 is an emerging chronic disease that has the potential to impact overall health and patients may experience mild to moderate symptoms including fatigue, chest pain, muscle pain, shortness of breath, cough, headache and “brain fog.” These symptoms last longer after infection with the Omicron variant of the SARS-CoV 2 virus (Thaweethai et al. 2023). However, there is a cumulative risk of post-acute sequelae, which may include various acute cardiac, pulmonary, or neurological and psychiatric symptoms. The incidence of long COVID-19 is increasing proportionally with the number of SARS-CoV-2 infections, especially in older people. Therefore, the number of cases of post-acute sequelae is expected to increase in the future (Boufidou et al. 2023).

Unfortunately, no effective approved treatment against Long COVID-19 has been discovered yet. The primary management of long-term COVID-19 currently relies on supportive treatment, symptomatic treatment and rehabilitation. A key part of the multidisciplinary approach to treatment involves the patient taking an active role in their recovery and self-monitoring (Schrimpf et al. 2022; Banerjee et al. 2022; Chee et al. 2023). Interestingly, the US National Institutes of Health (NIH) recently announced a series of clinical trials for potential treatments for long-term Covid-19 disease (Tanne 2023). There is now reasonable evidence that vaccination reduces the risk of long COVID-19. In a meta-analysis conducted in March 2023, people who received two doses of the vaccine were significantly less likely to develop long Covid-19 than unvaccinated people (Marshall 2023). However, reliable comparative studies, including randomized controlled trials, are needed to provide strong evidence of the efficacy of vaccination in preventing or relieving long COVID-19. A recent study observed a promising effect of metformin in preventing long-term COVID-19 disease compared to ivermectin or fluvoxamine. Outpatient treatment with metformin has been shown to reduce the incidence of long Covid by about 41%, compared to placebo. However, there was no significant effect of ivermectin or fluvoxamine on the cumulative incidence of long Covid compared to placebo (Bramante et al. 2023).

There are promising reasons to enhance research on the effects of vitamin D supplements in long COVID-19 patients. Several narrative reviews have indicated vitamin D as a mitigation agent for long COVID-19 and highlighted the potential role of hypovitaminosis D as a risk factor for long COVID-19 (Men´endez et al. 2022; Barrea et al. 2022; Moukayed 2023; Marks 2023). These publications are supported by the results of 4 observational studies published from January 2022 to Aug. 2023. However, no randomized controlled trial has been published to date that has found that vitamin D affects long COVID-19 syndrome. In one study, 681 post-COVID outpatient participants with long- COVID-19 were re-evaluated 6 months after hospital discharge. It was noted that vitamin D deficiency was detected in 35.6% of participants, and therefore vitamin D was an independent risk factor for long-COVID-19. Furthermore, vitamin D deficiency was associated with decreased performance with older participants (Galluzzo et al. 2022). In line with these findings, a previous study showed that COVID-19 survivors with vitamin D deficiency had lower exercise tolerance (Townsend et al. 2021).

In a retrospective case study, conducted from June 20 to July 31, 2022, blood concentrations of vitamin D, zinc, and fibrinogen were determined in patients infected with Omicron, a variant of the COVID-19 virus, who developed post-COVID-19 symptoms. This study showed that low serum VD level is associated with delayed recovery from long COVID-19 syndrome (Chen et al. 2023). In another retrospective cross-sectional study, fifty subjects with long COVID-19 and 50 subjects without long COVID-19 were enrolled on a 1:1 basis from the post-COVID-19 outpatient clinic. It was apparent that COVID-19 survivors with long COVID-19 had lower levels of VD 25(OH) than matched patients without long COVID-19 (di Filippo et al. 2023b). There is accumulating evidence to support the use of VD supplements, before and after infection with SARS-CoV-2, as a preventive strategy to reduce the risk of COVID-19, however, few other studies have reported a non-significant effect of VD in COVID-19. Metformin use may improve AMPK signaling of VDR and enhance VD efficacy in COVID-19 and long COVID-19. Randomized trials are needed to provide conclusive evidence of the effectiveness of VD or VD with metformin in preventing or ameliorating long COVID-19 disease in all patients.

## Genetic polymorphisms of the vitamin D could explain the controversy surrounding the clinical outcomes of VD supplementation

VD deficiency is highly prevalent worldwide and appears to be on the rise, and is common in critically ill patients (Xie et al. 2022; Cui et al. 2023). VD deficiency has been associated with a higher risk of severe COVID-19 infection (Dissanayake et al. 2022; Topan et al. 2023). However, the importance of VD supplementation in COVID-19 remains controversial. While some articles have observed a significant positive effect of VD on COVID-19 severity (ASLA et al. 2023; Petrelli et al. 2023), other studies have failed to find any benefit (Zhang et al. 2023). This controversy can be explained by differences in study design, samples studied, race, age, and genetic variations. Changes in serum 25(OH) D relative to VD supplementation vary widely among individuals. Recent genome studies have revealed associations of 25(OH)D concentration with proteins involved in vitamin D metabolism and transport. Variation in the expression and activities of these proteins could result in genetic differences in the production, transport and degradation of 25(OH) D that lead to differences in the level of 25(OH) D after vitamin D supplementation. Changes in the 25 (OH) D levels alter the dynamics and kinetics of VD. For example, increased endogenous production of vitamin D3 could be due to decreased enzymatic activity of 7-dehydrocholesterol reductase, encoded by the DHCR7 gene (Charoenngam et al. 2023).

Currently, several publications have addressed the relationship between genetic variation for vitamin D and treatment failure for COVID-19. Twenty-five articles on the association of genetic polymorphisms in the VDR or vitamin D binding protein (DBP) gene and individual responses to VD supplementation were published from January 2022 to August 2023. Several studies have indicated that there is significant variability in response to VD supplementation between people (Kelishadi et al. 2020; Ammar et al. 2023). This variability may result from a DBP polymorphism that contributes to the variability in the total plasma 25(OH) D concentrations of the VD supplementation. DBP gene encodes a DBP protein of 52 to 59 kDa, regulates absorption and plays important role in the transport of VD and its metabolites (Mehramiz et al. 2019; Slow et al. 2020; Ammar et al. 2023). This DBP polymorphism may explain the meta-analysis conclusion of Menger et al. (2022) about the superior effect on reducing COVID-19 mortality of parenteral VD supplementation. Other studies have indicated that gene polymorphisms in the VD metabolism pathway may alter susceptibility and severity of COVID-19 infection. Saria Santamira et al. (2023) observed an association of the CYP24A1 rs6127099 (A > T) polymorphism with a lower risk of COVID-19 infection. Furthermore, Foruhari et al. (2023) explain the variation of vitamin D levels across populations by epigenetic polymorphisms of CYP24A1 methylation.

Meanwhile, several studies noted that levels of 25(OH) D were not associated with the severity and mortality of COVID-19. These articles showed that response to VD supplementation can be altered by genetic variants of the VDR gene. The VDR genes are important for VD signaling and are modulated by genetic and non-genetic factors. Some VDR gene polymorphisms are independently linked with COVID-19 severity and patient survival (Ghiasvand et al. 2022; Apaydin et al. 2022; Jafarpoor et al. 2022). The TaqI variant allele and the FF variant FokI genotype can alter response to vitamin D supplementation and are associated with a better response (Usategui-Martín et al. 2022). Other researchers indicated that the mortality rate or severity of SARS-CoV-2 with different variants was associated with a low level of 25-OHD and that VDR SNPs regulate susceptibility to infection with COVID-19 (Camporesi et al. 2022; Al-Gharawi et al. 2023; Albu-Mohammed et al. 2022; Protas et al. 2023). Furthermore, it has been shown that patients with FokI and TaqI gene polymorphisms may be at higher risk of COVID-19 pandemic infection, and VD supplementation is recommended for individuals in the period surrounding or after the COVID-19 pandemic (Mamurova et al. 2023; Zeidan et al. 2023; Shawi et al. 2023)*.* It is clear from previous studies, VD deficiency and VDR polymorphisms are risk factors for COVID-19 and could explain the controversy surrounding the clinical outcomes of VD supplementation in the treatment of COVID-19. Large parenteral dose of VD supplementation may be recommended to reduce risk of DBP gene polymorphisms.

## Non-genetic reasons for differences in VD effects in COVID-19 and the use of metformin to improve VDR sensitivity

Unresponsiveness or VD resistance is not only caused by genetic disorders in vitamin D receptor expression, but impairment of vitamin D signaling (receptor resistance) may also be the cause as non-genetic mechanisms for variability of VD effects (Hampl and Vondra 2017; Macova et al. 2018). Metformin is the most frequently used first-line drug for the treatment of T2D. It attenuates insulin resistance or improves insulin receptor sensitivity through activation of AMPK or AMPK signaling and other mechanisms including several AMPK-independent mechanisms, such as affecting mitochondrial function, restoring redox homeostasis, and regulating several other signals, such as mTOR, SIRT1 and FBP1(Du et al. 2022). Several observations indicate that metformin is a unique multi-acting drug that targets multiple pathological pathways of COVID-19 in a diabetes-independent manner (Wiernsperger et al. 2022). Therefore, we hypothesize that metformin can improve VDR sensitivity through activation of AMPK or AMPK-independent mechanisms. This hypothesis is supported by preclinical, observational and clinical evidence that metformin may be beneficial in patients with acute, severe SARS-CoV-2 infection, long-COVID-19 and moderate evidence of benefit of metformin in preventing health care outcomes in COVID-19. In three clinical trials, metformin showed some effectiveness in protecting against severe cases of COVID-19 (Erickson et al. 2023).

In a comprehensive meta-analysis by Ganesh and Randall (2022), metformin improved outcomes in COVID-19 patients with DM and reduced mortality. In another meta-analysis including 22 retrospective observational studies, there was a significant association between reduced in-hospital mortality and outpatient metformin treatment for T2D in patients hospitalized for COVID-19 (Ma, Krishnamurth, 2023). Consistent with these studies, Pedrosa et al. (2023) examined the association between risk of death among COVID-19 patients and metformin use in 26 retrospective studies. They found that metformin use was significantly associated with a 13 to 90% reduction in death rate in patients with COVID-19. Importantly, a recent study showed that early use of metformin in treating COVID-19 outpatients reduced healthcare utilization for severe COVID-19 by 42.3% and the risk of long-term COVID by 41.3% over 10 months of follow-up( Bramante et al. 2023). Therefore, our study indicates a theoretical and practical basis for the use of metformin as a promising drug for improving VDR sensitivity, and its combination with vitamin D supplementation would be preferable in combating SARS-CoV-2 infection.

## Conclusion

There is accumulating evidence to support the use of VD supplements, before and after infection with SARS-CoV-2 (Long COVID-19), as a preventive strategy to reduce the risk of COVID-19 infection and mortality as well as prevent and treat post-COVID-19 syndrome. Several studies have indicated that early intramuscular administration of high-dose long-acting cholecalciferol had significantly better clinical outcomes in patients infected with COVID-19. However, few other studies with low-grade evidence have reported a non-significant effect of VD in COVID-19.This controversy surrounding the clinical outcomes of VD supplementation in the treatment of COVID-19 can be explained by VDR gene polymorphisms, DBP gene polymorphisms and impaired VDR signaling as non-genetic causes. Our study indicates a theoretical and clinical basis for the use of metformin as a promising drug to improve VDR sensitivity, and its combination with VD supplementation would be better in combating SARS-CoV-2 infection.

## Data Availability

Data on relevant human studies in the current review. https://docs.google.com/document/d/1d1BtamJEQzGDzQPtAR4O93-4Mx15hkQ7/edit?usp=sharing&ouid=113303836144481485524&rtpof=true&sd=true

## Abbreviations

AMPK: AMP activated protein kinase
ARDS: Acute respiratory distress Syndrome
ACE2: Angiotensin-converting enzyme 2
DBP: Vitamin D binding protein
COVID-19: Coronavirus disease 2019
25(OH) D2: Ergocalciferol; 1,25(OH)_2_ D3: cholecalciferol
CRP: C-reactive protein
DM: Diabetes mellitus
HMGB1: High-mobility group box 1 protein
iNOS: Inducible nitric oxide synthase
IL-1β: Interleukin one beta
IL-6: Interleukin-6
JAK: Janus kinase
JNK: C-Jun N-terminal kinase
LTB4: Leukotriene B4
mTOR: Mammalian target of rapamycin
PRKA: Protein kinase AMP-activated
NF-κB: Nuclear factor kappa B
RCT: Clinical controlled trial
SIRT1: Sirtuin
SARS-CoV2: Severe acute respiratory syndrome coronavirus2
TLR: Toll-like receptor 2
TNF-α: Tumor necrosis factor alpha
T2D: Diabetes type 2
VDR: Vitamin D receptor
